# Designer human tissue: coming to a lab near you

**DOI:** 10.1098/rstb.2017.0212

**Published:** 2018-05-21

**Authors:** David C. Hay, Cliona O'Farrelly

**Affiliations:** 1 MRC Centre for Regenerative Medicine, University of Edinburgh, 5 Little France Drive, EH16 4UU Edinburgh, UK; 2 Trinity Biomedical Sciences Institute, Trinity College Dublin, 152–160 Pearse St, Dublin 2, Dublin, Republic of Ireland

**Keywords:** self-renewal, differentiation, materials chemistry, bioengineering, label-free monitoring, cell-based therapy, cell-based modeling

## Abstract

Human pluripotent stem cells (PSCs) offer a scalable alternative to primary and transformed human tissue. PSCs include human embryonic stem cells, derived from the inner cell mass of blastocysts unsuitable for human implantation; and induced PSCs, generated by the reprogramming of somatic cells. Both cell types display the ability to self-renew and retain pluripotency, promising an unlimited supply of human somatic cells for biomedical application. A distinct advantage of using PSCs is the ability to select for genetic background, promising personalized modelling of human biology ‘in a dish’ or immune-matched cell-based therapies for the clinic. This special issue will guide the reader through stem cell self-renewal, pluripotency and differentiation. The first articles focus on improving cell fidelity, understanding the innate immune system and the importance of materials chemistry, biofabrication and bioengineering. These are followed by articles that focus on industrial application, commercialization and label-free assessment of tissue formation. The special issue concludes with an article discussing human liver cell-based therapies past, present and future.

This article is part of the theme issue ‘Designer human tissue: coming to a lab near you’.

The human body is composed of hundreds of different cell types that derive from pluripotent stem cells (PSCs). During development *in utero* and after birth, different stem cell populations perform vital functions in the body. These range from coordinated tissue morphogenesis during gestation, to tissue renewal and homeostasis in the adult. Essential to these processes is hierarchical control of cell potency. In the developing embryo and the adult, cell fate is determined by niche-specific factors and executed through defined changes in gene expression. A good example of cell fate determination is observed in PSCs, with the stem cell master regulators, Oct-4, Sox 2 and Nanog serving to instruct stem cell self-renewal and differentiation (for a review, see [1]).

Development starts with the fertilized egg cell, and *totipotent* blastomere formation, capable of forming embryonic and extra-embryonic tissues. At the point of blastocyst formation, the *pluripotent* cells of the inner cell mass are capable forming all three germ layers in the developing embryo, with the trophectoderm contributing to extra-embryonic tissues [2]. As development proceeds germ layer segregation takes place and multipotent stem cell populations are formed and differentiate into specialized tissues of the fetus. Following birth, *multi, bi* and *unipotent* stem cell populations persist in the neonate and the adult, serving to instruct development and/or tissue maintenance.

Since the 1980s, PSCs have taken centre stage as a promising cell candidate to model and treat human diseases. The successful isolation and culture of murine PSCs was presented in 1981 and heralded a new era in cell biology, driving important advances in our understanding of mammalian physiology [3,4]. It was a further 17 years before the first human embryonic stem cell lines were isolated [5]. These studies were pivotal to endeavours in modelling human physiology and developing cell-based therapies. The use of PSC-based systems will likely lead to new regulated cell-based assays in the near term, with tissue repair *in vivo* a longer-term aspiration. To date, a number of hESC-derived products are in clinical trials, including macular degeneration, diabetes and heart disease, with some other applications registered for clinical trial approval [6–8]. iPSCs have been used in the clinic, with one experimental procedure performed on an individual with macular degeneration [9]. Next year, clinical trials will commence using PSC-derived dopaminergic neurons to treat Parkinson's disease. The output from those well-controlled clinical trials will determine the suitability of PSCs for developing pioneering cell-based therapies. Although both types of PSCs are renewable, a distinct advantage of using iPSC-based systems is the ability to select for genetic background, promising personalized modelling of human biology ‘in a dish’ and/or immune-matched cell-based therapies for the clinic.

The landmark discovery that mammalian DNA, from a fully differentiated cell, could be reprogrammed to pluripotency leading to the birth of viable offspring [10] inspired the successful induction of pluripotency in mammalian somatic cells [11]. Reprogramming success was quickly extended to human somatic cells, providing a major advance for the field [12]. The successful isolation of PSCs and induction of pluripotency were deemed so important for basic research that two Nobel prizes in Physiology or Medicine were awarded in 2007 and 2012, respectively. The ability of PSCs to self-renew and form all cell types in the embryo proper is a distinct advantage and offers a scalable alternative to primary and transformed human tissue. Initially, stem cell reprogramming relied on retrovirus transduction to deliver transgene activity to the cells [11,12]. While this was important for proof of concept, retroviral and lentiviral transgene integration is problematic for basic or clinical research translation, therefore insertion-free strategies were developed (for a review, see [13]).

Since those seminal studies, there has been a global push to exploit PSC-based technology to challenge our understanding of human biology and to treat disease. Key outputs from these studies have been the generation of high-fidelity human models ‘in a dish’ and pioneering cell-based therapies to treat human disease. Stem cell-derived models have been developed for many tissues, including gut, liver and brain to name a few [14–16]. Stem cell-derived tissue systems have also been employed to study human disease [16], to study adverse drug reactions [17] and human susceptibility to viral infection [18]. Most recently, the development of efficient three-dimensional culture and organoid methodologies, combined with microfluidic platforms, have provided significant advances for the field, promising higher-fidelity datasets and perfused human tissue [19].

Annually, more than 115 million animals are used worldwide in experimentation [20]. While those experimental models provide valuable information, they do not efficiently extrapolate to human physiology, with an estimated 10% success rate [21]. This provides a strong rationale to develop more predictive human models. Consequently, there have been numerous studies focused on the delivery of defined and scalable human tissue systems. Key to producing high-fidelity tissue *in vitro* is the recreation of the nascent tissue structure, with the appropriate niche factors [22]. Attempts to achieve this *in vitro* and *in vivo* have seen scientists from chemistry, biology, physics and engineering backgrounds working together. This has led to exciting developments in human tissue engineering. With this in mind, we have prepared this special edition of the journal and invited contributions from experts around the world to describe cutting-edge activity in their fields and future directions.

We begin with a review of the progress made in the field of PSC reprogramming and self-renewal written by Abu-Dawud *et al*. [23]. This is followed by articles that examine the PSC somatic cell differentiation. The first article, written by McComish & Caldwell [24], focuses on brain differentiation. This is followed by an article reviewing gastrointestinal differentiation, written by Fair *et al*. [25]. The next article, written by Tam *et al*. [26], reviews musculoskeletal differentiation. We continue the somatic cell differentiation theme with articles focusing on the immune system. Macrophage differentiation from iPSCs is presented by Lopez-Yrigoyen and co-workers [27]. This is followed by an article on the innate immune system written by Fischer and co-workers [28]. Following on from somatic cell differentiation, Lyall and co-workers [29] present the use of a semi-automated differentiation system to model fatty liver disease.

Essential to the generation of cell-based models and therapies is the quality of the *in vitro* engineered tissue. Underpinning success in technology scale-up and application is the development of the raw materials required to produce the somatic cells at clinical grade. A key consideration is the extra-cellular matrix used in the production process and this is reviewed by Hagbard and co-workers [30]. Recently, the use of technologies that examine global patterns of gene expression facilitated major improvements in cell and phenotype and scale-up [31,32]. Such advances are also necessary to assure the quality of PSC-based products. The use of bioinformatics to control stem cell differentiation is reviewed by Godoy and co-workers [33]. This is followed by an article by Meisig & Blüthgen [34] dealing with the deconstruction of cell signalling equilibria to build bona fide tissue from PSCs.

In recent years, tremendous progress has been made in the development of organoids from human tissue [35,36]. These studies are exciting, but for organoids to offer significant promise they must be manufactured at scale, using defined additives. Developments in this field of research are reviewed by Alhaque and co-workers [37]. In order to deliver engineered tissue at scale, it will be necessary to further optimize the cell niche and automate the production process. Materials chemistry provides a unique opportunity to identify new materials that can be used to structure, scale and stimulate appropriate tissue formation *in vitro* [38]. Recent progress in the field is reviewed by Schmidt and co-workers [39]. Following on from this, Skeldon and co-workers [40] discuss the importance of cell bioprinting in the quest to generate human tissue. This is followed by Brown & Khetani reviewing the microfabrication of stem cell-derived tissue for cell-based screening [41]. Stem cell-derived somatic cells have already shown significant promise within industry [17]. For these models to progress further, there are further technological considerations. This is dealt with in an opinion piece article, written by Williams from Astra Zeneca [42].

The ability to produce high-fidelity human tissue from stem cells for basic and clinical application requires the development of non-invasive monitoring technology that can measure and report in real time. The merits of label-free monitoring are reviewed by Gamal and co-workers [43]. We end this special issue with an article by Iansante and co-workers [44], which reviews the progress made in developing human cell-based therapies to treat compromised liver function and promote organ regeneration.

We are extremely grateful to the authors, the reviewers and the editorial team at Philosophical Transactions B for their time and effort in delivering this special edition of the journal. We hope that this collection of papers stimulates interest and collaboration within the field, with a focus on translating basic scientific concepts into game-changing regenerative medicines for the clinic.

## Data accessibility

This article has no additional data.
